# Resveratrol stereoselectively affected (±)warfarin pharmacokinetics and enhanced the anticoagulation effect

**DOI:** 10.1038/s41598-020-72694-0

**Published:** 2020-09-28

**Authors:** Tse-Yin Huang, Chung-Ping Yu, Yow-Wen Hsieh, Shiuan-Pey Lin, Yu-Chi Hou

**Affiliations:** 1 Ph.D. Program for Biotech Pharmaceutical Industry, School of Pharmacy, China Medical University, Taichung, 40402 Taiwan, ROC; 2 School of Pharmacy, China Medical University, Taichung, 40402 Taiwan, ROC; 3 Department of Pharmacy, China Medical University Hospital, Taichung, 40447 Taiwan, ROC

**Keywords:** Cardiovascular diseases, Preclinical research

## Abstract

Resveratrol (RVT) has various beneficial bioactivities and popularly used as a dietary supplement. RVT showed inhibitions on CYP1A2/2C9/3A4, breast cancer resistance protein (BCRP), and some conjugated metabolites of RVT also inhibited BCRP. (±)Warfarin, an anticoagulant for cardiovascular disease but with narrow therapeutic window, were substrates of CYP1A2/3A4(R-form), 2C9(S-form) and BCRP. We hypothesized that the concurrent use of RVT might affect the metabolism and excretion of warfarin. This study investigated the effect of RVT on the pharmacokinetics and anticoagulation effect of (±)warfarin. Rats were orally given (±)warfarin (0.2 mg/kg) without and with RVT (100 mg/kg) in a parallel design. The results showed that RVT significantly increased the AUC_0−t_ of S-warfarin and international normalized ratio. Mechanism studies showed that both RVT and its serum metabolites (RSM) inhibited BCRP-mediated efflux of R- and S-warfarin. Moreover, RSM activated CYP1A2/3A4, but inhibited CYP2C9. In conclusion, concomitant intake of RVT increased the systemic exposure of warfarin and enhanced the anticoagulation effect mainly via inhibitions on BCRP and CYP2C9.

## Introduction

**Figure 1:**
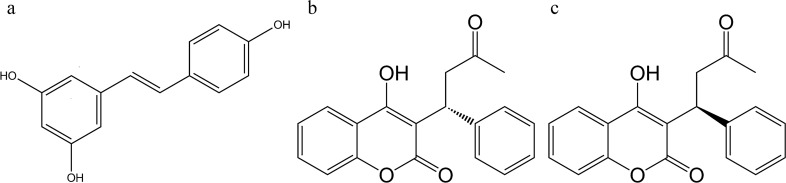
Chemical structures of resveratrol (**a**), R-warfarin (**b**) and S-warfarin (**c**).

Resveratrol (RVT), a polyphenolic stilbene constituent (chemical structure shown in Fig. 1) in grapes, berries and peanuts, etc., has shown various bioactivities, such as antioxidation^1^, anti-inflammation^2^ and antiaging^3^. Owing to the benefits of RVT and grape extract to human cardiovascular diseases^4–6^, RVT is nowadays a popular dietary supplement. Although RVT was believed to be harmless to healthy individuals^7,8^, it showed inhibitions on metabolic enzymes such as CYP1A2, 2C9, CYP3A4^9^ and transporters such as P-glycoprotein (P-gp)^10^, breast cancer resistance protein (BCRP)^11^*.* Furthermore, RVT enhanced the ototoxicity of cisplatin in rats^12^ and increased the anticoagulation effect of warfarin in mice^13^. Therefore, we suspected that concurrent use of RVT might affect the pharmacokinetics of various medicines, and probably result in alteration of the efficacy and safety of critical drugs.

Warfarin, an important anticoagulant, has been widely prescribed to treat patients with cardiovascular diseases such as deep vein thrombosis and pulmonary embolism, but with narrow therapeutic window^14^. In clinical practice, the anticoagulation effect of warfarin is routinely maintained by frequent monitoring the international normalized ratio (INR) to ensure the safety and efficacy of treatment. Warfarin was administered in a racemic form consisting of equal amounts of R- and S-warfarin (chemical structures shown in Fig. 1)^15^. S-warfarin was 2.7–3.8 times more potent than R-form^16^. In pharmacokinetic aspect, S-warfarin was mainly metabolized by CYP2C9, whereas R-warfarin was metabolized by CYP1A2 and CYP3A4^15,17,18^. About 80% of an oral dose of warfarin was excreted in urine^19^. Recently, warfarin was verified as a substrate of BCRP^20^, an efflux transporter locating at the apical side of intestine ^21^ and proximal tubule^22^.

In regard to the metabolic fate of RVT, extensive and rapid transformation to its glucuronides and sulfates was reported^23^. Among the metabolites, RVT glucuronides were substrates of BCRP^24^, in addition, resveratrol-3-glucuronide, resveratrol-3-sulfate and resveratrol-di-sulfate were inhibitors of BCRP^25^. We herein hypothesized that RVT and its conjugated metabolites might inhibit the BCRP-mediated efflux transport of warfarin at intestine and proximal renal tubule. Therefore, this study investigated the effect of concomitant intake of RVT on the pharmacokinetics and anticoagulation effect of warfarin in rats. Furthermore, the underlying mechanisms were clarified via in vitro and *ex-vivo* approaches.

## Results

### Quantitation of RVT in the capsule powders

The HPLC chromatogram of powder extract revealed a satisfactory resolution (Supplementary Fig. 1) of RVT and methylparaben (internal standard). Quantitation result indicated that the capsule powders contained 985 mg/g of RVT.

### Effect of RVT on the pharmacokinetics of R- and S-warfarin

**Figure 2:**
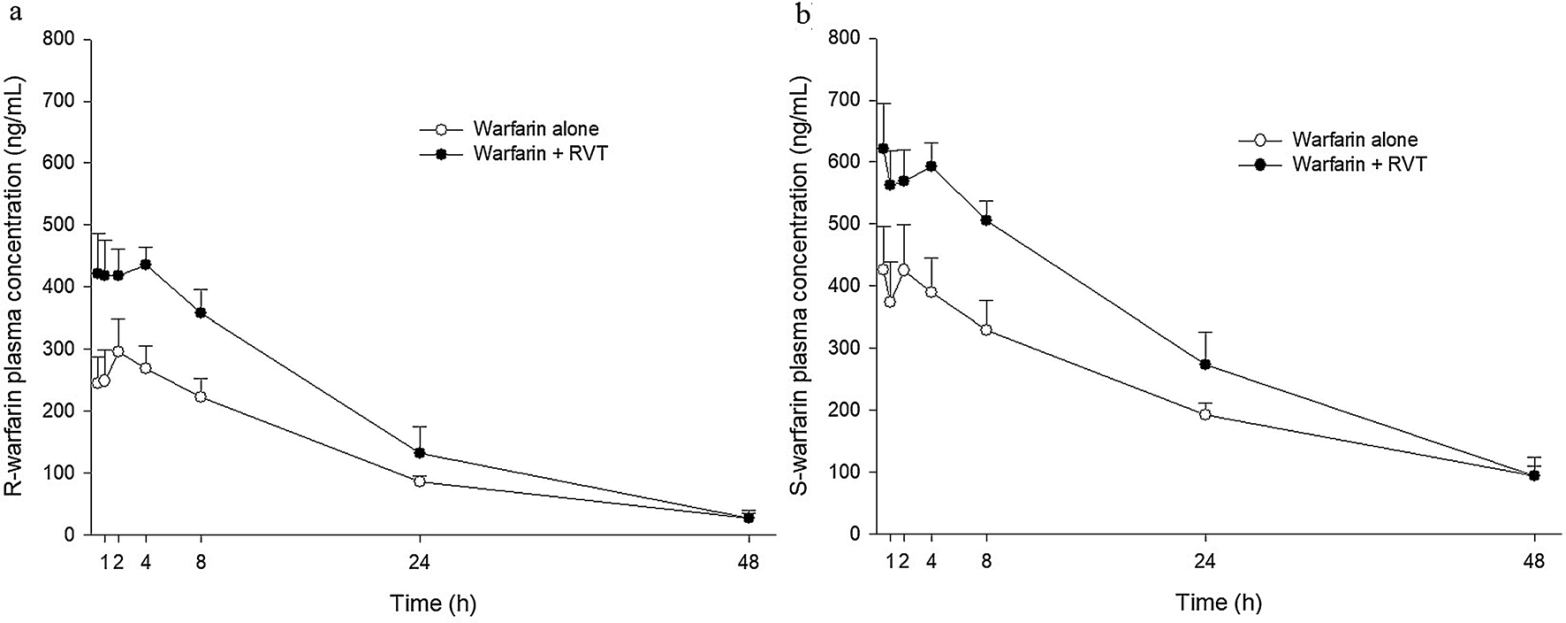
Mean (± SEM) plasma concentration–time profiles of R-warfarin (**a**) and S-warfarin (**b**) after oral administration of warfarin alone and co-administration with RVT (100 mg/kg) at 0.5 h before warfarin dosing. Each group included 8 rats.

The plasma concentration–time profiles of R- and S-warfarin in rats after oral administration of warfarin alone and coadministration of RVT at 0.5 h before warfarin are shown in Fig. 2a,b, respectively. The pharmacokinetic parameters of R- and S-warfarin after two treatments are listed in Table 1. The results showed that coadministration of RVT significantly increased the AUC_0−24_ of both R-warfarin and S-warfarin by 58.6% and 48.8%, respectively. The AUC_0−t_ of S-warfarin was significantly increased by 42.1%, whereas the increase of R-warfarin by 55.1% did not reach statistical significance (*p* = 0.07).

**Table 1:** Pharmacokinetic parameters of R-warfarin and S-warfarin after oral administration of warfarin (0.2 mg/kg) alone and co-administration with RVT (100 mg/kg) at 0.5 h before warfarin. Each group included 8 rats.

Parameter	Treatment	
	Warfarin alone	Warfarin + RVT
**R-warfarin**		
C_max_ (ng/mL)	315.1 ± 53.4	482.7 ± 49.0
AUC_0−24_ (h·ng/mL)	4471.1 ± 546.4	7089.3 ± 842.6* + 58.6%
AUC_0−t_ (h·ng/mL)	5758.8 ± 672.0	8931.2 ± 1466.5 + 55.1% (*p* = 0.07)
MRT_0−t_ (h)	13.4 ± 1.1	11.6 ± 1.2
**S-warfarin**		
C_max_ (ng/mL)	489.5 ± 76.1	672.0 ± 61.0
AUC_0−24_ (h·ng/mL)	7128.3 ± 837.6	10,605.7 ± 829.4* + 48.8%
AUC_0−t_ (h·ng/mL)	10,563.9 ± 1054.5	15,009.5 ± 1749.0* + 42.1%
MRT_0−t_ (h)	17.2 ± 0.9	15.0 ± 1.0

Data expressed as mean ± SEM *p < 0.05.

C_max_, maximum concentration.

AUC_0−24_, area under concentration–time curve from 0 to 24 h.

AUC_0−t_, area under concentration–time curve to the last time.

MRT_0−t_, mean residence time from the time of dosing to the time of last measurable concentration.

### Effect of RVT on the anticoagulation effect of warfarin

**Figure 3:**
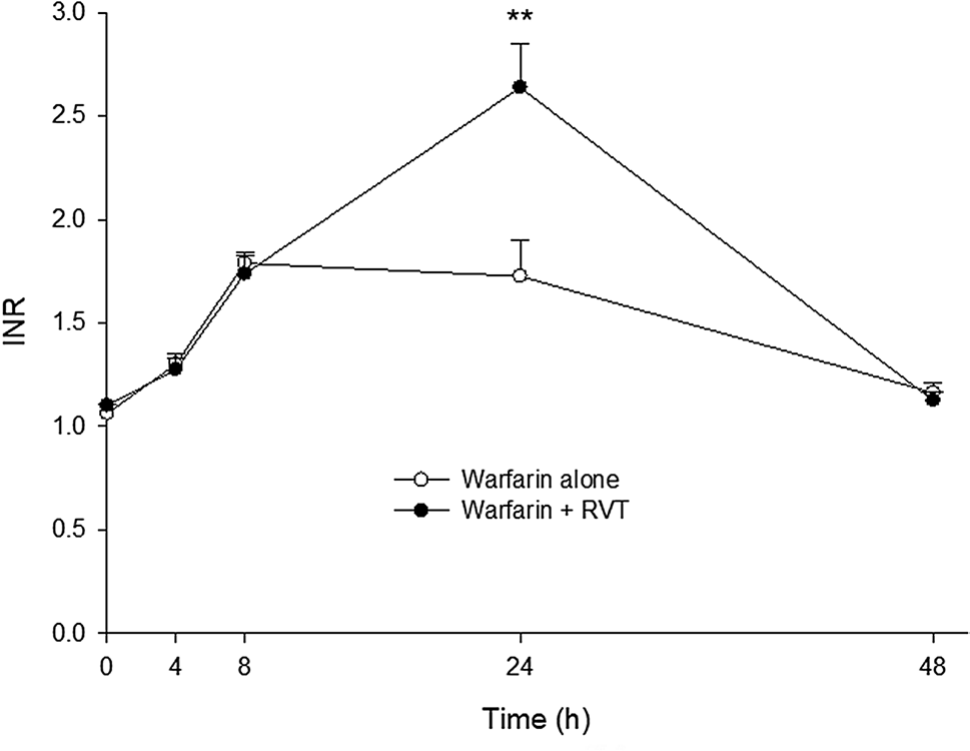
Mean (± SEM) INR—time profiles after oral administration of warfarin (0.2 mg/kg) alone and co-administrations with RVT (100 mg/kg) at 0.5 h before warfarin. Each group included 8 rats. **p < 0.01.

Figure 3 shows the INR-time profiles of rats after a dose of warfarin alone and with a dose of RVT before warfarin. The INR measured at 24 h after warfarin dosing of rats treated with warfarin alone (1.73 ± 0.18) was significantly lower than that of rats coadministered with RVT (2.64 ± 0.21).

### Characterization of RSM

After enzymatic hydrolysis and quantitative analysis by HPLC, the result indicated that the RSM prepared from rats contained 1.9 μM of RVT and 57.9 μM of RVT sulfates/glucuronides.

### Effect of RVT on the efflux transport of R- and S-warfarin

**Figure 4:**
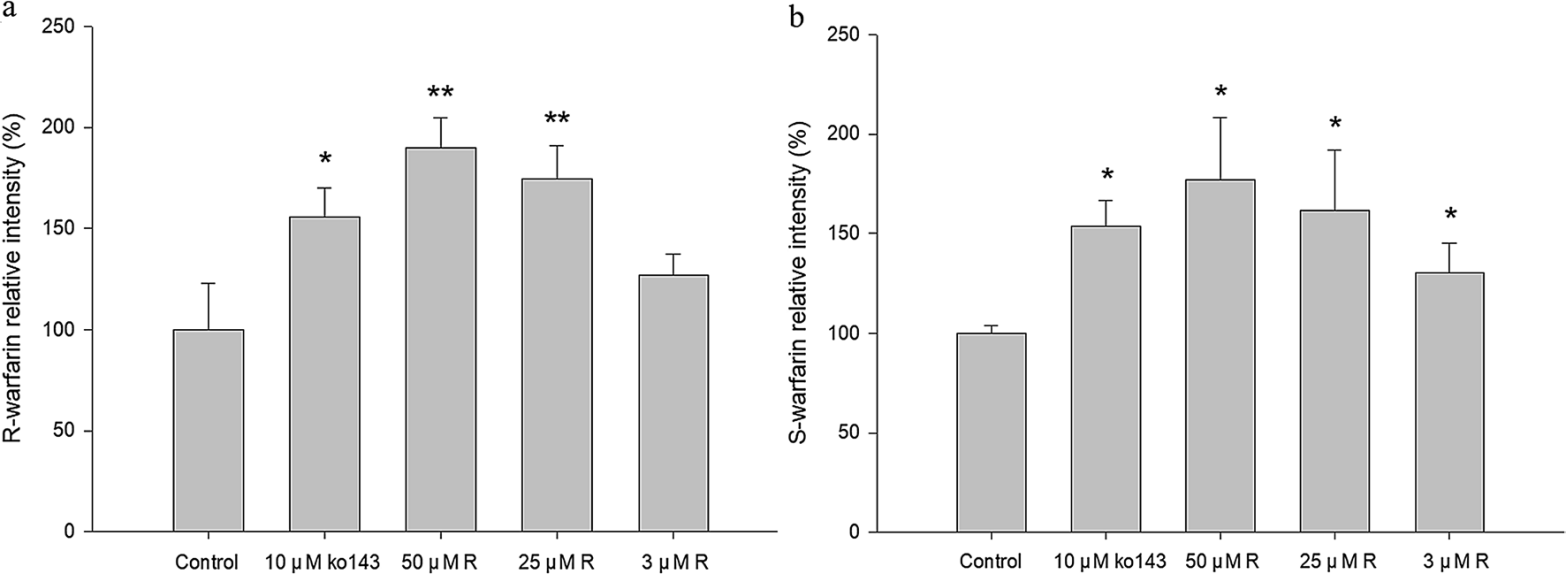
Effect of RVT on the intracellular accumulation of R-warfarin (**a**) and S-warfarin (**b**) in MDCKII-BCRP cells. Data expressed as mean ± SD.*p < 0.05, **p < 0.01.

The cell viability of MDCKII-BCRP was not affected after 1-h incubation with RVT (100 μM). The relative intracellular accumulations of R- and S-warfarin after incubation with and without RVT or Ko143 (10 μM) are shown in Fig. 4a, b, respectively. The results showed that 50 μM and 25 μM of RVT significantly increased the intracellular accumulations of R-warfarin by 90.0% and 74.3%, respectively, and 50 μM, 25 μM and 3 μM of RVT significantly increased the intracellular accumulations of S-warfarin by 77.0%, 61.7% and 30.0%, respectively. As a positive control, Ko143 (10 μM) increased the intracellular accumulations of R- and S-warfarin by 55.6% and 53.7%, respectively.

### Effect of RSM on the efflux transport of R- and S-warfarin

**Figure 5:**
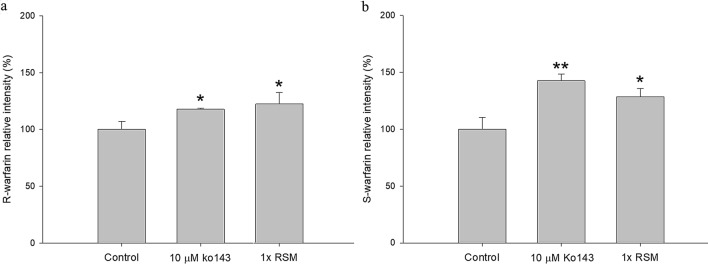
Effect of RSM at onefold serum concentration on the intracellular accumulation of R-warfarin (**a**) and S-warfarin (**b**) in MDCKII-BCRP cells. Data expressed as mean ± SD. *p < 0.05, **p < 0.01.

The cell viability of MDCKII-BCRP was not affected after 1-h incubation with RSM (onefold serum concentration). The relative intracellular accumulations of R- and S-warfarin after incubation with and without RSM (onefold serum concentration) or Ko143 (10 μM) are shown in Fig. 5a, b, respectively. The RSM at onefold serum concentration significantly increased the intracellular accumulation of R- and S-warfarin by 22.4% and 28.5%, respectively. As a positive control in onefold blank serum metabolite, Ko143 significantly increased the intracellular accumulations of R- and S-warfarin by 17.7% and 42.5%, respectively.

### Effect of RSM on the activities of CYP1A2, 2C9 and 3A4

**Figure 6:**
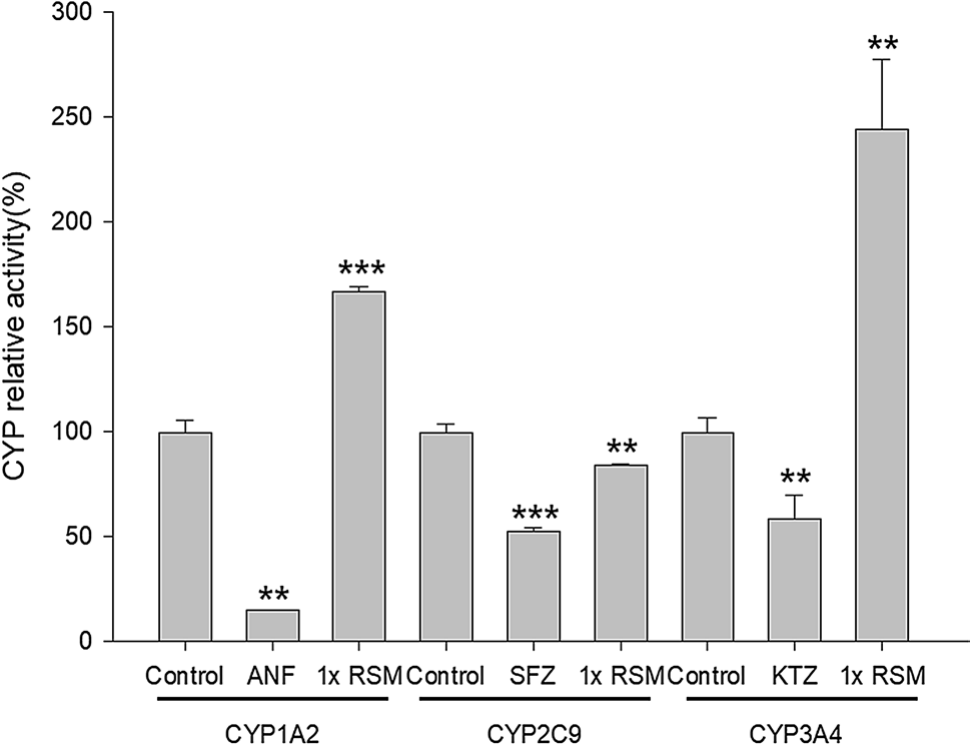
Effects of blank serum (control) and RSM at onefold serum concentration on the activities of CYP1A2, CYP2C9 and CYP3A4. ANF, α-naphthoflavone, a positive control of CYP 1A2 inhibitor; KTZ, ketoconazole, a positive control of CYP 3A4 inhibitor. SFZ, sulfaphenazole, a positive control of CYP 2C9 inhibitor. Data expressed as mean ± SD. *p < 0.05, **p < 0.01, ***p < 0.001.

Figure 6 showed that RSM at onefold serum concentration significantly decreased the activity of CYP2C9 by 15.7%, conversely, the activities of CYP1A2 and CYP3A4 were significantly increased by 66.8% and 144.5%, respectively. The inhibition effects of CYPs inhibitors were partially blocked by the blank serum but remained significant (Supplementary Fig. 2). ANF (10 μM), SFZ (30 μM) and KTZ (30 μM) significantly decreased the activities of CYP1A2, CYP2C9 and CYP3A4 by 85.1%, 47.5% and 41.2%, respectively.

## Discussion

In the present pharmacokinetic study, our results showing that a single dose of RVT given prior to warfarin significantly increased the AUC_0−24_ of both R- and S-warfarin indicated that RVT increased the absorption of both enantiomers of warfarin. As to the total systemic exposure, the AUC_0−t_ of S-warfarin was significantly increased by 42.1%, but the increase of AUC_0−t_ of R-warfarin by 55.1% did not reach statistical significance (*p* = 0.07), which might be due to insufficient animal number.

The results of pharmacodynamic study showed that a single dose of RVT given prior to warfarin significantly elevated the INR of rats at 24 h post warfarin dosing, revealing that RVT increased the anticoagulation effect of warfarin, that echoed the result of a recent study in mice^13^. Importantly, our above-mentioned pharmacokinetic study could explain the enhanced anticoagulation effect by increased systemic exposure of S-warfarin, the more potent enantiomer.

Whether the increased anticoagulation effect was arisen from RVT itself? Although RVT had been reported to disrupt the coagulation by inhibiting the aggregation of platelets^23,26^, INR was not affected in the RVT-fed mice^13^. In order to confirm this fact, 4 rats were given 7 repeated doses of RVT (100 mg/kg, thrice daily) and our result showed that RVT alone did not increase the INR (Supplementary Table 1), indicating that the increased INR in this interaction study was not due to the bioactivity of RVT itself. We in turn speculated that RVT increased the anticoagulation effect of warfarin through pharmacokinetic interaction.

Concerning the mechanisms underlying RVT–warfarin pharmacokinetic interaction, our in vitro results showing that RVT significantly increased the intracellular accumulation of R- and S-warfarin in MDCKII-BCRP cells indicated that RVT inhibited the BCRP-mediated efflux transport of R- and S-warfarin. Moreover, RSM at onefold serum concentration likewise increased the intracellular accumulation of R- and S-warfarin, indicating that RSM like RVT inhibited the BCRP-mediated efflux transport of R- and S-warfarin. Taken together, both RVT and RSM inhibited BCRP, which might lead to decreased excretion of warfarin at gut lumen and proximal renal tubule.

Regarding another probable mechanism concerning CYPs, although previous in vitro studies had shown that RVT inhibited CYP1A2^27^, CYP2C9 and CYP3A4^9,28^, we considered these results could not be extrapolated to in vivo situation because of the extensive metabolism of RVT^23^. Therefore, we had prepared RSM from rats in order to mimic the major molecules interacting with CYPs in the enterocytes and hepatocytes. Characterization of RSM indicated that the major molecules were the glucuronides/sulfates of RVT (57.9 μM), and the parent form of RVT was very minor (1.9 μM, about 3%). The evaluation of modulations on CYPs showed that RSM inhibited CYP2C9, the major metabolic enzyme of S-warfarin, but activated CYP1A2 and CYP3A4, the major metabolic enzymes of R-warfarin, indicating that the intake of RVT exerted opposite modulations on the metabolism of R- and S-warfarin. Accordingly, our results clearly revealed that the pharmacokinetic influence of RVT on (±)warfarin was stereoselective. The inhibition of RSM on CYP2C9 could in part account for the significant increase of the systemic exposure of S-warfarin. In addition, our study disclosing activations on CYP1A2 and CYP3A4 by RSM were apparently opposite to the reported inhibition by the parent form of RVT^9,28^.

Taken together, our mechanism studies showed that both RVT and RSM inhibited BCRP, in addition, RSM inhibited CYP 2C9, but activated CYP 1A2 and CYP 3A4. Therefore, the increased systemic exposure of S-warfarin could be accounted for by the decreased BCRP-mediated excretion and inhibited CYP2C9-catalyzed metabolism, which exerted synergistic effect and might lead to bleeding.

Despite of various health benefits of RVT, warfarin-treated patients should be cautious for the hidden risk of bleeding when taking RVT as a dietary supplement. We suggest that patients taking long-term medications, especially high-alert medicines with narrow therapeutic window should be aware of the probable interactions between RVT and drugs.

In conclusion, concomitant intake of RVT increased the systemic exposure of warfarin and enhanced the anticoagulation effect mainly via inhibitions on BCRP and CYP2C9.

## Materials and methods

### Chemicals and reagents

(±)Warfarin sodium (purity 98%), caffeic acid (purity 98%), formic acid, 3-(40,50-dimethylthiazol-20-yl)-2,5-diphenyltetrazolium bromide (MTT) and sodium dodecyl sulfate were purchased from Sigma-Aldrich Chemical Co. (MO, U.S.A.). R-warfarin (purity 98%) and S-warfarin (purity 98%) were obtained from Toronto Research Chemicals, Inc. (Ontario, Canada). Ko143 (purity 96%) was obtained from Enzo Life Sciences, Inc. (NY, U.S.A) Fetal bovine serum, Dulbecco’s modified Eagle medium, penicillin–streptomycin-glutamine and Hank’s buffered salt solution (HBSS) were obtained from Invitrogen (CA, U.S.A). Resveratrol (purity > 99%) was obtained from TCI (Tokyo, Japan). Trans-resveratrol capsules were purchased from Doctor’s BEST (CA, U.S.A.). Acetonitrile and methanol of LC–MS grade were obtained from J.T. Baker Inc. (PA, U.S.A.).

### HPLC–UV, LC–MS apparatus and chromatographic conditions

The analysis of resveratrol was performed by using an LC-10AT VP (Shimadzu Corporation, Kyoto, Japan) equipped with an SLC-10A system controller, an SIL-10AF auto injector and an SPD-10A UV/visible detector. The separation was performed by using an Apollo C18 5μ column (W.R. Grace and Company, U.S.A.).

The LC–MS analysis of R- and S-warfarin followed the method used in a previous study^20^. Briefly, the separation was carried out by using a chiral column Astec Chirobiotic V (Sigma-Aldrich Chemical Co., U.S.A.) and detection was performed under negative ionization mode with selected ion monitoring of mass-to-charge ratio (m/z) of 307 for R- and S-warfarin, and 179 for caffeic acid.

### Quantitation of RVT in the nutraceutical powders

Powders decanted from Trans-resveratrol Capsules were dissolved with 70% methanol, centrifuged under 10,000 g for 15 min, and the supernatant was analyzed by HPLC–UV. Methylparaben was used as internal standard, and the mobile phase consisting of methanol (A) and 0.1% phosphoric acid (B) with A/B = 50/50 was run isocratically. The detection wavelength was set at 280 nm.

### Animals, drug administration and blood collection

Male Sprague–Dawley rats were purchased from BioLASCO Taiwan Co., Ltd. (Taipei, Taiwan). The animal study adhered to “Guideline for the care and use of laboratory animals” published by the Council of Agriculture Executive Yuan, Taiwan, R.O.C. and the experimental protocol had been approved by the Institutional Animal Care and Use Committee of China Medical University, Taiwan.

For pharmacokinetic study, 16 rats (440–560 g) were randomly divided into two groups and fasted overnight before experiments. Control rats were orally administered (±)warfarin (0.2 mg/kg) via gastric gavage. The rats in experimental group were orally administered (±)warfarin (0.2 mg/kg) with a dose (100 mg/kg) of Trans-resveratrol Capsule powders suspended in H_2_O (containing 50 mg/mL of resveratrol) at 0.5 h before warfarin in a parallel design. Food was offered 3 h after warfarin dosing. Blood samples (0.6 mL) were collected at 0.5, 1, 2, 4, 8, 24, 48 h after warfarin dosing and collected in Vacutainer K2 EDTA tube (BD, NJ, U.S.A.).

For pharmacodynamic study, 16 rats (440–600 g) were randomly divided into two groups and drug administration was the same as that described in the above-mentioned pharmacokinetic study. Blood samples (50 µL) were collected at 0, 4, 8, 24 and 48 h after warfarin dosing, and INR was measured by using CoaguChek XS System (Roche Diagnostics GmbH, Mannheim, Germany).

### Preparation and characterization of RVT serum metabolites (RSM)

Nineteen rats were pretreated with Trans-resveratrol Capsule powders (100 mg/kg) thrice daily. After the 6th dose, rats were fasted for 12 h and then given the 7th dose. Blood was collected at 20 min after the 7th dose. After coagulation, the serum was mixed with fourfold volume of methanol and centrifuged at 10,000*g* for 15 min. The supernatant was concentrated in a rotatory evaporator under vacuum to dryness. An appropriate volume of ddH_2_O was added to afford a specimen with tenfold serum concentration, which was divided into 1.0-mL aliquot per vial and freeze-dried. Each vial was added with water to afford desired concentration before use.

The characterization of RSM followed a previous method^28^. Free form of resveratrol was quantified by HPLC–UV. The resveratrol glucuronides/sulfates were indirectly quantified through hydrolysis. Briefly, 100 μL of serum metabolite specimen was mixed with 200 μL of sulfatase (containing 1000 unit/mL of sulfatase and > 14,830 unit/mL β-glucuronidase) in pH 5 buffer, 50 μL of ascorbic acid (100 mg/mL) and incubated at 37 °C for 1 h. The mixture was acidified with 50 μL of 0.1 N HCl and partitioned with 400 μL of ethyl acetate (containing ethylparaben 50 μg/mL as internal standard). After centrifugation, the ethyl acetate layer was dried by nitrogen gas and reconstituted with 50 μL of methanol, and 5 μL was subject to HPLC–UV analysis. The conditions of HPLC followed that described previously for the quantitation of resveratrol in nutraceutical powders.

### Cell lines and culture conditions^20^

Human BCRP-transfected Madin-Darby canine kidney II cells (MDCKII-BCRP) were kindly provided by Prof. Dr. Piet Borst (Netherlands Cancer Institute, Amsterdam, Netherlands). Cells were cultured in Dulbecco’s modified Eagle medium supplemented with 10% fetal bovine serum and 1% penicillin–streptomycin-glutamine. Cells were maintained at 37 °C in a humidified incubator containing 5% CO_2_. The medium was changed every other day and cells were subcultured when 80% to 90% confluency was reached.

### Effects of RVT and RSM on the viability of MDCKII-BCRP cells

The cell viability of MDCKII-BCRP was evaluated by MTT assay^20^. After seeding the cells onto 96-well plates for overnight incubation, 100 μM of resveratrol and onefold serum concentration of RSM were added and incubated for 1 h, then MTT was added and incubated for additional 4 h. Sodium dodecyl sulfate solution (10%) was added to lyse the cells, and then the cell viability was determined at 570 nm using a microplate reader (BioTek, Winooski, VT).

### Effect of RVT on the efflux transport of R- and S-warfarin

MDCKII-BCRP cells were seeded onto 12-well plates at a density of 3 × 10^5^ cells per well. After 3-day culturing, the medium was removed and washed with ice-cold phosphate-buffered saline (PBS). All cells were firstly pre-incubated with 400 μL of R- or S-warfarin (15 μM in pH 7.4 HBSS) at 37 °C for 60 min, then washed with ice-cold PBS, and further incubated with 400 μL of resveratrol (50, 25 and 3 μM), 1% dimethyl sulfoxide and Ko143 (10 μM, as positive control) for 20 min. Finally, cell lysates were obtained after trypsinization with 500 μL of 0.25% Trypsin–EDTA and lyzed by liquid nitrogen.

### Effect of RSM on the efflux transport of R- and S-warfarin

MDCKII-BCRP cells were seeded onto 12-well plates at a density of 3 × 10^5^ cells per well. After 3-day culturing, the medium was removed and washed with ice-cold PBS. Cells were incubated with 400 μL of R- and S-warfarin (15 μM) with or without Ko143 (10 μM, as positive control) in onefold serum concentration of blank serum metabolite or RSM for 30 min. Finally, cell lysates were obtained after trypsinization with 500 μL of 0.25% Trypsin–EDTA and lyzed by liquid nitrogen.

### Quantitation of R- and S-warfarin in plasma and cell lysate^20^

Plasma sample (100 µL) was mixed with 50 µL of 0.5 N formic acid and partitioned with 150 µL of ethyl acetate containing 10 µg/mL of caffeic acid as internal standard. After centrifugation, the ethyl acetate layer was dried by nitrogen gas and reconstituted with methanol (50 µL), and 5 µL was subject to LC–MS analysis.

The cell lysate (400 µL) was added 50 µL of 0.5 N formic acid and partitioned with 450 µL of ethyl acetate containing 1 µg/mL of caffeic acid as internal standard. After centrifugation, the ethyl acetate layer was dried by nitrogen gas and reconstituted with methanol (50 µL), and 5 µL was subject to LC–MS analysis.

### Effect of RSM on the activities of CYP1A2, 2C9 and 3A4

Vivid CYP450 screening kits (Thermo Fisher Scientific Inc.) were used to evaluate the effect of RSM on the activities of CYP1A2, 2C9, and 3A4. All the procedures were performed following the manual provided by the manufacturer. Briefly, after incubating 2.5-fold serum concentration (diluted to onefold serum concentration when incubating with CYPs substrates) of RSM with CYP450 recombinant BACULOSOMES, glucose-6-phosphate and glucose-6-phosphate dehydrogenase in a 96-well black plate at room temperature for 10 min, plate was pre-read. Then, specific CYP substrates (Vivid EOMCC Substrate for CYP1A2, Vivid OOMR Substrate for CYP2C9 and Vivid BOMR Substrate for CYP3A4) and NADP^+^ were added for further 30-min incubation. Finally, 0.5 M Tris-base was added to stop the reaction. The fluorescence was measured by SpectraMax iD3 Multi-Mode Microplate Reader (Molecular devices, USA) with excitation at 415 nm (CYP1A2) and 530 nm (CYP2C9 and CYP3A4) and emission at 460 nm (CYP1A2) 590 nm (CYP2C9 and CYP3A4).α-naphthoflavone (ANF), sulfaphenazole (SFZ) and ketoconazole (KTZ) were spiked in blank serum and treated as positive controls of CYP1A2, CYP 2C9, and CYP3A4, respectively.

### Data analysis

The pharmacokinetic parameters were calculated by Phoenix WinNonlin version 7.0 (Pharsight Corporation, St. Louis MO, USA). Unpaired Student’s t-test was used for statistical comparisons taking *p* < 0.05 as significant.

## Supplementary information

Supplementary information

## Abbreviations

BCRP: Breast cancer resistance protein
CYP: Cytochromes P450
HBSS: Hank’s buffered salt solution
INR: International Normalized Ratio
KTZ: Ketoconazole
MDCKII: Madin–Darby canine kidney type II cells
MTT: 3-(40,50-Dimethylthiazol-20-yl)-2,5-diphenyltetrazolium bromide
ANF: α-Naphthoflavone
PBS: Phosphate-buffered saline
P-gp: P-glycoprotein
RVT: Resveratrol
RSM: Resveratrol serum metabolites
SFZ: Sulfaphenazole
