# Supplementary material for: Resveratrol stereoselectively affected (±)warfarin pharmacokinetics and enhanced the anticoagulation effect
Source: Sci Rep. 2020 Sep 28;10:15910. doi: 10.1038/s41598-020-72694-0 (PMC7522226; doi:10.1038/s41598-020-72694-0)
Supplement: Supplementary file 1 — Supplementary information [file 41598_2020_72694_MOESM1_ESM.docx]

**Resveratrol stereoselectively affected (±)warfarin pharmacokinetics and enhanced the anticoagulation effect**

Tse-Yin Huang^a,1^, Chung-Ping Yu^b,c,1^, Yow-Wen Hsieh^b,c^, Shiuan-Pey Lin^b,*^ and Yu-Chi Hou^b,c,*^

^a^ *Ph.D. Program for Biotech Pharmaceutical Industry, School of Pharmacy, China Medical University, Taichung, 40402, Taiwan, ROC.*

^b^ *School of Pharmacy, China Medical University, Taichung, 40402, Taiwan, ROC.*

^c^ *Department of Pharmacy, China Medical University Hospital, Taichung, 40447, Taiwan, ROC.*

^1^ These authors contributed equally to the study

^*^ For corresponding author

Dr. Yu-Chi Hou and Shiuan-Pey Lin, School of Pharmacy, China Medical University, 91, Hsueh-Shih Road, Taichung, 40402, Taiwan, ROC. Tel and Fax: 886-4-22031028

*E-mail addresses*: hou5133@gmail.com (Y.-C. Hou) and splin@mail.cmu.edu.tw (S.-P. Lin)


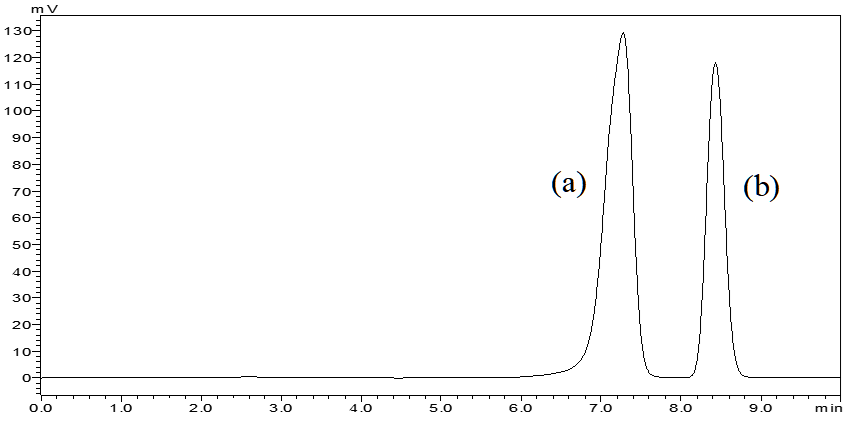
Supplementary Fig. 1 HPLC-UV chromatogram of resveratrol and methylparaben in methanol. (a) Resveratrol and (b) methylparaben (internal standard).


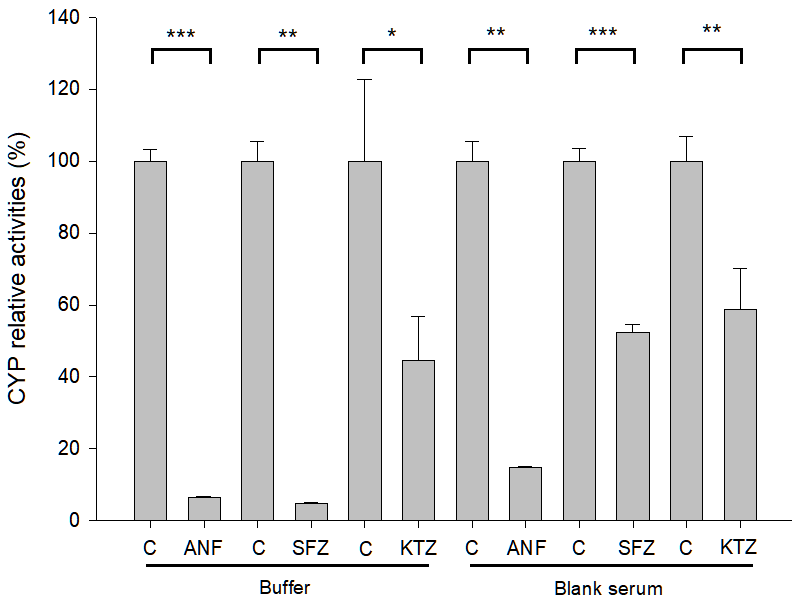


Supplementary Fig. 2 Inhibition effects of CYPs inhibitors in buffer and blank serum. ANF,α-naphthoflavone, a CYP 1A2 inhibitor; C, control; KTZ, ketoconazole, a CYP 3A4 inhibitor. SFZ, sulfaphenazole, a CYP 2C9 inhibitor. Data expressed as mean ± SD. * p<0.05, ** p<0.01, *** p<0.001.


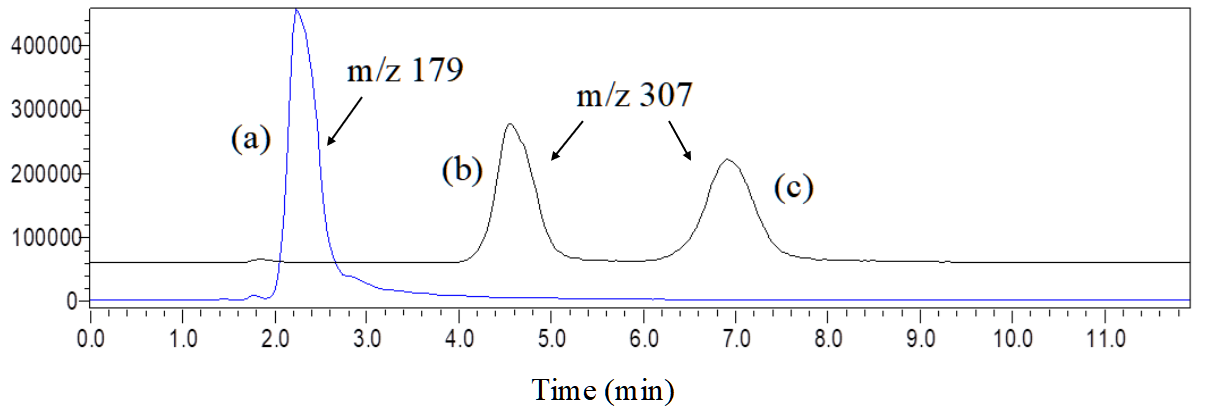


Supplementary Fig. 3 HPLC-MS chromatogram of R-/S-warfarin in methanol. (a) caffeic acid (internal standard, m/z 179), (b) R-warfarin (m/z 307), and (c) S-warfarin (m/z 307)

| Rats  Time | 1 | 2 | 3 | 4 |
| --- | --- | --- | --- | --- |
| Before resveratrol dosing | 1.0 | 1.0 | 1.0 | 1.0 |
| After 7 doses of resveratrol | 1.0 | 1.0 | 1.0 | 1.0 |

Supplementary Table 1 Raw INR data of resveratrol-fed rats before and after 7 doses of resveratrol (100mg/kg, blood samples collected at 20 minutes after the 7^th^ dose).
